# Investigating the Influence of Ribavirin on Human Respiratory Syncytial Virus RNA Synthesis by Using a High-Resolution Transcriptome Sequencing Approach

**DOI:** 10.1128/JVI.02349-15

**Published:** 2016-04-29

**Authors:** Waleed Aljabr, Olivier Touzelet, Georgios Pollakis, Weining Wu, Diane C. Munday, Margaret Hughes, Christiane Hertz-Fowler, John Kenny, Rachel Fearns, John N. Barr, David A. Matthews, Julian A. Hiscox

**Affiliations:** a Institute of Infection and Global Health, University of Liverpool, Liverpool, United Kingdom; b Health Protection Research Unit in Emerging and Zoonotic Infections, Liverpool, United Kingdom; c Centre for Genomic Research, University of Liverpool, Liverpool, United Kingdom; d School of Medicine, Boston University, Boston, Massachusetts, USA; e School of Molecular and Cellular Biology, University of Leeds, Leeds, United Kingdom; f School of Cellular and Molecular Medicine, University of Bristol, Bristol, United Kingdom

## Abstract

Human respiratory syncytial virus (HRSV) is a major cause of serious respiratory tract infection. Treatment options include administration of ribavirin, a purine analog, although the mechanism of its anti-HRSV activity is unknown. We used transcriptome sequencing (RNA-seq) to investigate the genome mutation frequency and viral mRNA accumulation in HRSV-infected cells that were left untreated or treated with ribavirin. In the absence of ribavirin, HRSV-specific transcripts accounted for up to one-third of total RNA reads from the infected-cell RNA population. Ribavirin treatment resulted in a >90% reduction in abundance of viral mRNA reads, while at the same time no such reduction was detected for the abundance of cellular transcripts. The presented data reveal that ribavirin significantly increases the frequency of HRSV-specific RNA mutations, suggesting a direct influence on the fidelity of the HRSV polymerase. The presented data show that transitions and transversions occur during HRSV replication and that these changes occur in hot spots along the HRSV genome. Examination of nucleotide substitution rates in the viral genome indicated an increase in the frequency of transition but not transversion mutations in the presence of ribavirin. In addition, our data indicate that in the continuous cell types used and at the time points analyzed, the abundances of some HRSV mRNAs do not reflect the order in which the mRNAs are transcribed.

**IMPORTANCE** Human respiratory syncytial virus (HRSV) is a major pediatric pathogen. Ribavirin can be used in children who are extremely ill to reduce the amount of virus and to lower the burden of disease. Ribavirin is used as an experimental therapy with other viruses. The mechanism of action of ribavirin against HRSV is not well understood, although it is thought to increase the mutation rate of the viral polymerase during replication. To investigate this hypothesis, we used a high-resolution approach that allowed us to determine the genetic sequence of the virus to a great depth of coverage. We found that ribavirin did not cause a detectable change in the relative amounts of viral mRNA transcripts. However, we found that ribavirin treatment did indeed cause an increase in the number of mutations, which was associated with a decrease in virus production.

## INTRODUCTION

Human respiratory syncytial virus (HRSV) is one of the major lower respiratory tract pathogens, and almost all infants are infected at least once within the first 2 years of life (1). Elderly patients, patients with chronic heart and lung conditions, and immunocompromised patients are also at risk (2–4). According to the World Health Organization, approximately 60 million people are infected with HRSV every year, resulting in up to 160,000 deaths (5).

HRSV belongs to the genus Pneumovirus of the family Paramyxoviridae in the order Mononegavirales (6, 7). The viral genome consists of a nonsegmented, ∼15-kb RNA of negative polarity that encodes 10 mRNAs and 11 proteins. As with all the members of the Mononegavirales, the genomic RNA of HRSV is tightly bound by the viral nucleoprotein (N) and maintained as a helical N-RNA ribonucleoprotein (RNP) complex (8). The RNP is used as a template for transcription and replication by the RNA-dependent RNA polymerase (RdRp) complex, which consists of the large subunit (L) and its cofactor phosphoprotein (P) (9, 10). Whereas N, P, and L are sufficient to mediate viral replication, transcription activity also requires the M2-1 protein, which functions as an RdRp processivity cofactor (11, 12). The specific recognition of the viral N-RNA complex by the RdRp constitutes a prerequisite for viral transcription and replication. This recognition is mediated by the P protein (13), which interacts with the L, N, and M2-1 proteins.

To perform transcription and replication, the RdRp engages with promoter sequences that lie at the 3′ ends of the genome and antigenome RNAs (14). The 44-nucleotide (nt) leader (Le) promoter region at the 3′ end of the genome is responsible for directing initiation of mRNA transcription and antigenome synthesis. The 155-nt trailer complement promoter at the 3′ end of the antigenome directs genome RNA synthesis. The 10 viral genes are arranged sequentially (3′-NS1, NS2, N, P, M, SH, G, F, M2, and L-5′), and each is flanked by conserved gene start (GS) and gene end (GE) sequences, which control the RdRp during transcription (15, 16). Each gene is separated from the preceding one by an intergenic region of variable length. The RdRp initiates RNA synthesis at the leader promoter and then progresses along the length of the genome (16–18). The RdRp is able to generate subgenomic mRNAs by responding to the GE and GS signals at the gene junctions. When it reaches a GE signal, the RdRp polyadenylates and releases the nascent mRNA of the upstream gene. It then reinitiates mRNA synthesis of the subsequent gene at the next GS signal. There is a tendency for the RdRp to cease transcription at the gene junctions, and on rare occasions, the transcribing RdRp fails to respond to a GE signal, resulting in synthesis of a polycistronic mRNA that contains an intergenic region. Because the RdRp can initiate transcription only at the 3′ end of the genome and can disengage transcription at the gene junctions, it is expected that there is a gradient of expression of HRSV mRNAs, with genes at the 3′ end of the genome (e.g., NS1 and NS2) being transcribed more frequently than genes at the 5′ end of the genome (e.g., the L gene), and indeed this has been shown to be the case *in vitro* (19).

Ribavirin is the only therapeutic approved by the Food and Drug Administration (FDA) for the treatment of HRSV (20). Clinically, ribavirin is used in immunocompromised and/or transplant and acute high-risk groups infected with HRSV (for example, see reference 21). Ribavirin has broad-spectrum antiviral properties and is also used clinically in the treatment of infections with hepatitis E virus (HEV) (22) and hepatitis C virus (HCV) (23) and for diseases caused by several hemorrhagic fever viruses (for example, see reference 24). Ribavirin is a purine nucleoside analog which is metabolized to ribavirin triphosphate by cellular kinases (25, 26). Ribavirin (and its phosphorylated derivatives) has been shown to have multiple effects that may enable its broad-spectrum antiviral activity, including, among others, enhancement of interferon-stimulated gene expression; inhibition of the cellular enzyme inosine 5′-monophosphate dehydrogenase (IMPDH), which is required for maintenance of the intracellular pool of GTP; chain termination during viral RNA synthesis; inhibition of 5′-methylguanosine cap formation; and accumulation of mutations in viral genomes (reviewed in references 27 to 29). In the latter case, mutations accumulate because ribavirin is capable of base pairing equally well with cytidine and uridine, resulting in an increase of the rate of C-to-U and G-to-A transitions (30–32). This results in hypermutation, which can be lethal to virus biology through error catastrophe. This mechanism of action has been proposed for and supported by experimental data *in vitro* for poliovirus (33) and HCV and *in vivo* for HCV (23).

Previous studies have investigated the effect of IMPDH inhibition during HRSV infection (34, 35), but the effect that ribavirin might have on the fidelity of HRSV RNAs or the stability of resulting mRNA transcripts has not previously been examined. To investigate the influence of ribavirin on HRSV RNA synthesis, we used high-resolution transcriptome sequencing (RNA-seq). Minor variant analysis allowed us to assess the effect of ribavirin on the frequency of mutations in the HRSV genome. The addition of ribavirin resulted in a decrease in the abundance of viral RNA and a modest increase in the frequency of transition but not transversion mutations, suggesting a direct influence on polymerase fidelity. In addition, we found that in both the absence and presence of ribavirin, the cumulative abundances of viral mRNAs at the two time points analyzed did not follow the transcription gradient of mRNA synthesis, contrary to what was anticipated.

## MATERIALS AND METHODS

### Cells and virus.

HEp-2 cells were grown at 37°C with 5% CO_2_ in Dulbecco's modified Eagle's medium (DMEM) supplemented with 10% (vol/vol) fetal bovine serum (FBS) and 1% (vol/vol) penicillin-streptomycin. The HRSV-A2 strain was used in this study and was purified through a sucrose gradient prior to the infection experiments. The virus was not plaque purified and thus was heterogeneous.

### Antiviral ribavirin.

Ribavirin was obtained from Sigma. A 1 mM stock was preprepared in dimethyl sulfoxide (DMSO) and added at either 6 or 24 h postinfection (hpi). MTT [3-(4,5-dimethyl-2-thiazolyl)-2,5-diphenyl-2H-tetrazolium bromide] assays were performed to ensure that cell viability was not affected at the working concentration.

### Immunofluorescence.

Coverslip-adhered cell monolayers were fixed with paraformaldehyde and made permeable with phosphate-buffered saline (PBS) containing 0.1% (vol/vol) Triton before adding a fluorescein isothiocyanate (FITC)-conjugated anti-HRSV-A2 specific primary antibody from Abcam (ab20391).

### Western blotting.

Cell lysates were prepared, and the total protein concentration was determined by bicinchoninic acid (BCA) assay (Pierce). Proteins (5 μg/μl) were resolved by 7.5% SDS-PAGE and transferred to polyvinylidene difluoride (PVDF) membranes (Millipore) by use of a Bio-Rad semidry transfer apparatus.

### Determination of 50% tissue culture infective dose (TCID_50_).

HEp-2 cells were seeded in 24-well plates at a density of 5 × 10^4^ in 1 ml growth medium in order to have ∼80% confluence following overnight incubation. The medium was aspirated from the wells after 24 h, and cells were washed twice with PBS. Extracellular and intracellular preparations (200 μl) were added to 4 wells of 96-well plates (HEp-2 cells at 80% confluence). The plates were incubated for 2 h, with shaking every 20 min. The inoculum was removed, and 200 μl of maintenance medium was added and incubated for 7 days. At 7 days postinfection, all plates were examined, and the number of wells of each dilution that were positive for an HRSV cytopathic effect (CPE) (syncytium formation) was counted and calculated by using the Kärber algorithm.

### Determining cell viability.

Cell viability was determined by using a colorimetric MTT assay. HEp-2 cells were seeded in triplicate in 96-well plates at a density of 1 × 10^4^ to ensure 70% confluence at 24 h, after which all wells were washed twice with 1× PBS. Ribavirin was then added to wells at different concentrations (low to high) and incubated for 24 h at 37°C. Next, the wells were washed twice with 1× PBS. MTT (0.024 g; Sigma) was prepared in 10 ml of medium (DMEM plus 10% FBS for HEp-2 cells) and warmed to 37°C. The MTT was dissolved and filtered, 100 μl was added to each well, and the plate was incubated for 50 min at 37°C. After this incubation, the MTT was removed, 100 μl of DMSO was added and mixed, and the color was measured at 570 nm by use of a Tecan reader.

### RNA extraction and RNA sequencing.

Total RNA was extracted from HEp-2 cells (in triplicate) by use of a Qiagen RNeasy minikit. The RNA concentration was measured by the Qubit RNA BR assay. Agarose gel electrophoresis with ethidium bromide (EtBr) staining was used to visualize RNA in order to check the quality of each RNA sample. cDNA was synthesized from RNA via reverse transcription in order to check the amplification by using an RdRp chain reaction. Samples were quality checked for integrity (Bioanalyzer RNA picochip) and quantity (Qubit RNA kit). After assessing that the RNA quality was good, with RNA integrity number (RIN) values of >9, the samples were subjected to poly(A) selection by use of a Dynabeads mRNA purification kit (Life Technologies). In this case, 15 μl of beads per sample was washed with binding buffer (2×) and resuspended in 30 μl of 2× bead buffer. Samples were made up to 30 μl and heated to 65°C for 5 min to remove any secondary structure. Samples were mixed with the beads on a rotator for 5 min at room temperature. After collecting the beads on a Magnetic Particle Concentrator (MPC), the beads were washed twice with wash buffer, with removal of all traces after each wash step. Twelve microliters of water was added to the beads for elution, which was achieved by heating the sample at 70°C for 2 min and retrieving the supernatant containing the RNA. This was assessed for rRNA on a RNA picochip.

The twice-selected material was all used as input for ScriptSeq assay (Epicentre). Samples were treated per the manufacturer's protocol. Samples were mixed with primer and fragmentation buffer, heated at 85°C for 5 min, and placed on ice. Samples were converted to cDNA and purified with Ampure XP. Samples were mixed with bar-coded primers and amplified with 15 PCR cycles. The libraries were purified with Ampure XP and assessed by use of a Bioanalyzer and an HS DNA kit, and the quantity was determined by use of a Qubit DNA HS kit. Samples were pooled on an equimolar basis for a single lane.

The quantity and quality of the final pool were assessed using a Qubit kit (Invitrogen) and a Bioanalyzer (Agilent) and then quantitative PCR, using a Kapa library quantification kit on a Roche LC480II Light Cycler machine according to the manufacturer's instructions. The template DNA was denatured according to the protocol described in the Illumina cBot user guide and loaded at 9 pM. To improve the sequencing quality control, 1% PhiX174 was spiked into the sample. Sequencing was carried out on an Illumina HiSeq 2000 instrument with version 3 chemistry, generating 100-bp paired-end reads.

For each time point, the sequence reads were mapped to the HRSV-A2 genome by using Bowtie2. For each alignment to the HRSV genome, the output BAM files were further analyzed using QuasiRecomb (36). While Bowtie2 aligned more than 1 million reads to the HRSV genome, we selected 1 million alignments at random. We used the coverage option for QuasiRecomb (command line java − XX:NewRatio = 9 − Xms2G − Xmx10G − jar QuasiRecomb.jar − i reads.sam − coverage) and then parsed the output files with in-house software written in Perl that determined the most abundant nucleotide for each position (i.e., the consensus nucleotide) and reported the frequency of use for the other three nucleotides, as described previously (37).

## RESULTS

Various studies have revealed the inhibitory nature of ribavirin on HRSV *in vitro*. For example, addition of ribavirin at −2, 0, and 1 hpi resulted in a 95% plaque reduction (38); however, the mechanism of this inhibition is unclear.

RNA sequencing has not been applied at a high read depth to study HRSV replication and transcription, and it provides an ideal approach to study the potential mutagenic effect of ribavirin on HRSV genome biology. We utilized our recently described pipeline (based on the Galaxy platform), which was used to generate consensus genomes and maps of minor variants to study Ebola virus evolution in a guinea pig model (39) and in patient samples taken from the 2014-2015 West African outbreak (37). These studies indicated that a greater read depth during RNA-seq provided more accurate base calling on the consensus genome and maps of minor variants.

**FIG 1:**
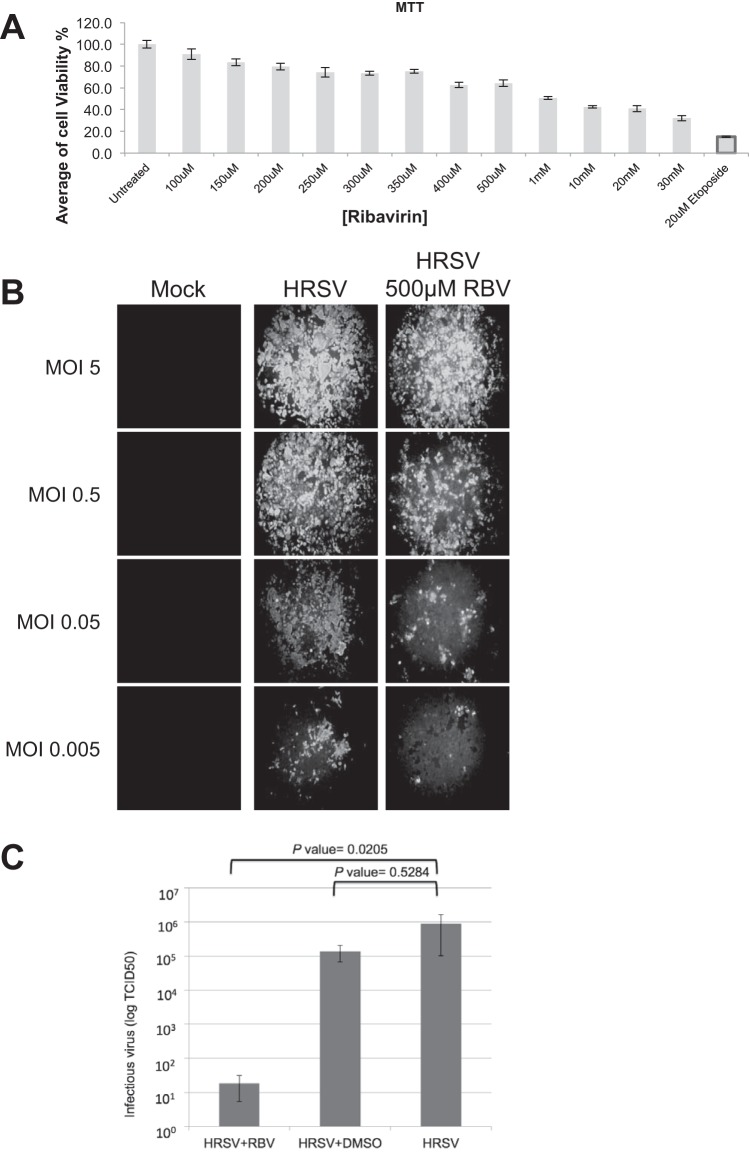
Analysis of HRSV in the absence and presence of ribavirin. (A) An MTT assay was used to determine cell viability in the presence of ribavirin. Etoposide was used as a positive control. (B) Immunofluorescence was used to evaluate HRSV-infected cells at different MOIs and in the absence and presence of 500 μM ribavirin (RBV). (C) Progeny virus production in the presence and absence of RBV and in the DMSO-only control.

To provide sufficient viral RNA for sequencing and to balance this with infectivity and cell viability (through both treatment with ribavirin and infection with HRSV), several optimization experiments were performed. The concentration of ribavirin used was based on previous literature, and an MTT assay was used to establish cell viability versus toxicity in HEp-2 cells (Fig. 1A). These analyses, together with concentrations previously used in the literature, suggested that a concentration of 500 μM would be tolerated and provide antiviral activity. Next, we investigated four different viral multiplicities of infection (MOIs) (0.005, 0.05, 0.5, and 5) in the absence and presence of 500 μM ribavirin by using a fluorescence-based assay system to visualize HRSV-infected cells (Fig. 1B). These data indicated that at an MOI of 0.5 and in the presence of 500 μM ribavirin, there was a visible decrease in the number of infected cells compared to untreated cells. At a lower MOI, fewer cells were infected (therefore providing less RNA for robust sequencing). The inhibitory action of ribavirin on the output of HRSV progeny was confirmed using the TCID_50_. This indicated that the amount of progeny virus was reduced approximately 5 log in the presence of 500 μM ribavirin compared to either untreated infected cells or infected cells treated with the DMSO vehicle.

**FIG 2:**
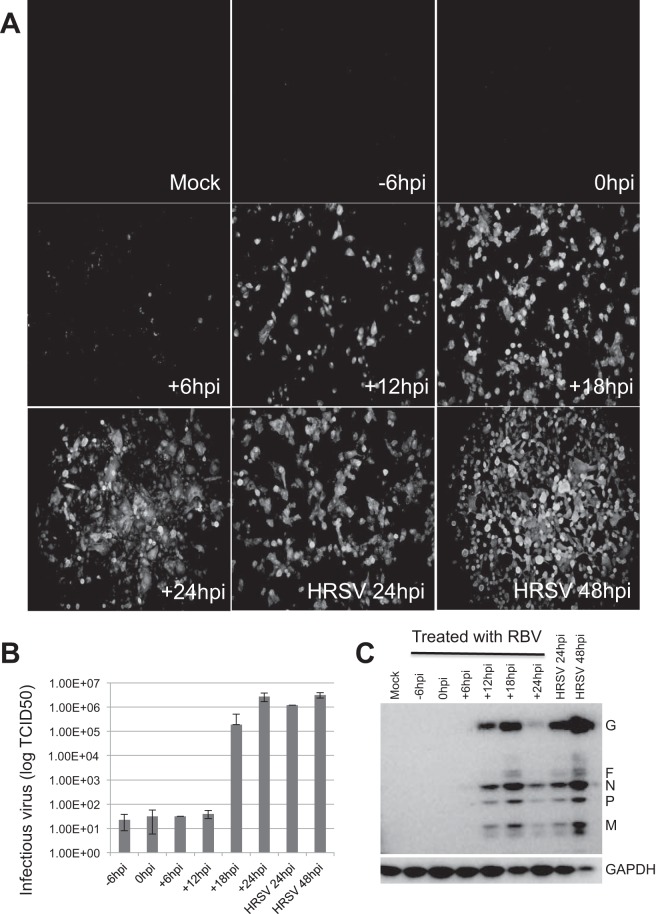
Comparison of HRSV biology in response to different ribavirin (RBV) treatment regimens. Cells were treated with ribavirin at −6, 0, 6, 12, 18, or 24 hpi. For treatment with ribavirin at −6, 0, 6, 12, and 18 hpi, an assay point of 24 hpi was used, and for treatment with ribavirin at 24 hpi, an assay point of 48 hpi was used. Immunofluorescence (A), TCID_50_ (B), and Western blot (C) assays were used to evaluate virus-infected cells, viral progeny production, and viral protein abundance, respectively. In panel C, the species corresponding to each viral protein is identified. GAPDH, glyceraldehyde-3-phosphate dehydrogenase.

Next, the effects of ribavirin treatment at −6, 0, 6, 12, 18, and 24 hpi on HRSV biology were compared (Fig. 2). For treatment with ribavirin at −6, 0, 6, 12, and 18 hpi, an assay point of 24 hpi was used, and for treatment with ribavirin at 24 hpi, an assay point of 48 hpi was used. Immunofluorescence, TCID_50_ determination, and Western blotting were used to evaluate virus-infected cells (Fig. 2A), viral progeny production (Fig. 2B), and viral protein abundance (Fig. 2C), respectively. On the basis of these findings, in order to ensure sufficient read depth and to derive information on sequence changes, the following time points were chosen for further analysis: (i) ribavirin added at 6 hpi and RNA harvested at 24 hpi and (ii) ribavirin added at 24 hpi and RNA harvested at 48 hpi.

### Determining the abundances of viral mRNAs in HRSV-infected cells by using RNA-seq.

RNA-seq was used to measure the abundances of poly(A)-selected viral mRNAs at the two different time points in both untreated and ribavirin-treated HEp-2 cells. RNA-seq analysis for the two different time points indicated that in untreated HEp-2 cells at 24 and 48 hpi, 19.8% and 10.53% of total reads mapped to HRSV RNA (Table 1), corresponding to 6,162,832 and 3,810,092 reads, respectively. HRSV transcripts also reached a high level in A549 cells, in this case representing 34.72% of the total reads (Table 1), for a total of 14,105,224 reads.

**TABLE 1:** Numbers and proportions of sequence reads mapping to the HRSV genome out of the total number of sequence reads

Cell line	Conditions	No. of reads mapping to the HRSV genome	Total no. of reads	% of total reads
HEp-2	HRSV at 24 hpi	6,162,832	31,118,416	19.8
	HRSV + ribavirin at 24 hpi	21,350	32,769,666	0.07
	HRSV at 48 hpi	3,810,092	36,182,362	10.53
	HRSV + ribavirin at 48 hpi	436,172	44,545,058	0.98
A549	HRSV at 24 hpi	14,105,224	40,622,791	34.72
	HRSV + 17-AAG at 24 hpi	1,549,050	37,493,536	4.13

**FIG 3:**
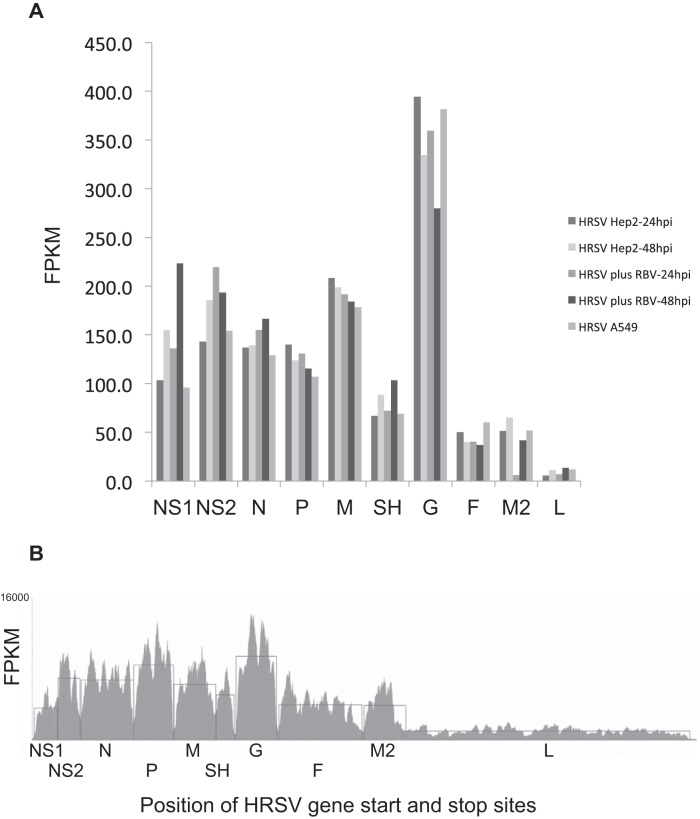
(A) Analysis of the abundances of viral mRNAs in HEp-2 or A549 cells infected with HRSV at different time points and in HEp-2 cells in the absence or presence of ribavirin. The viral mRNA abundances are shown in order of the particular genes along the HRSV genome, listed from the 3′ to the 5′ end, and also in the order in which the particular genes are transcribed. Abundance is shown in fragments per kilobase of exon per million fragments mapped (FPKM). (B) RNA-seq data for HEp-2 cells at 24 hpi were further processed to include only proper mate pair reads, where both reads were mapped and correctly orientated with respect to each other. The seed length was increased from 28 to 35 nt. The maximum number of mismatches was reduced to 1. The horizontal lines trace the average abundances of mapped reads for the different genes.

From the RNA-seq data, we calculated the relative abundances of each viral mRNA at 24 and 48 hpi in the absence and presence of ribavirin treatment by using the numbers of reads mapping to each gene. We were not able to discriminate monocistronic viral mRNAs from polycistronic RNAs, although the abundances of polycistronic readthrough products are generally low in HRSV infections (40). The data indicated that as a proportion of total reads, the amount of viral RNA decreased in the presence of ribavirin (Table 1). The accumulation of each mRNA in an infected cell might be expected to reflect the order of genes on the genome, such that the relative abundance of different gene transcripts would correlate with gene position relative to the 3′ end. However, for the two time points examined, the data showed that while there was a general trend of abundance correlating with gene order, there were exceptions (Fig. 3A). For example, as expected, the L mRNA was the least abundant transcription product (Fig. 3A), but the mRNA encoding the G protein appeared to be the most abundant mRNA at both time points in both the absence and presence of ribavirin. This observation suggests that factors other than gene order may be able to influence HRSV mRNA abundance in cells, in particular for the G mRNA. Importantly, the profiles of relative viral mRNA abundance were equivalent for untreated and ribavirin-treated infected HEp-2 cells, suggesting that ribavirin did not impinge upon the overall program of HRSV transcription *per se*.

We hypothesized that if viral mRNA transcript levels are governed in part by mRNA stability, the profile of relative levels might be different in different cell types. Therefore, we investigated the abundances of viral mRNAs in HRSV-infected A549 cells at 24 hpi. In this instance, 34.72% of the total reads mapped to the HRSV mRNAs (Table 1), for a total of 14,105,224 reads. Again, the data indicated that the abundances of viral mRNAs at this time point and in this cell type did not correspond to the predicted linear gradient (Fig. 3A).

To investigate this further, as an exemplar, the RNA-seq data for HRSV in HEp-2 cells at 24 hpi were further processed to include only proper mate pair reads, where both reads were mapped and correctly orientated with respect to each other. The seed length was increased from 28 to 35 nt. The maximum number of mismatches was reduced to 1. One effect of this was to bias the reads toward those that matched the consensus sequence. These data again indicated that the L mRNA was the least abundant HRSV transcript (Fig. 3B); however, the differences in the other gene products were less pronounced, with P and G transcripts being almost equivalent (Fig. 3B).

### Readthrough at gene junctions correlates with previous subgenomic replicon data.

The data also provided a measurement of readthrough, which allowed a direct comparison to previously published results. This was done by comparing the average number of fragments per kilobase of exon per million fragments mapped (FPKM) in the intergenic region to the total virus FPKM. From this, the percentages of readthrough between adjacent genes were calculated, with the following results: 30% for NS1-NS2, 13% for NS2-N, 10% for N-P, 4% for P-M, 21% for M-SH, 4% for SH-G, 8% for G-F, and 11% for F-M2. For the gene junctions examined, this pattern of relative readthrough efficiencies correlates with data examining the transcription termination efficiencies of gene junctions in the context of subgenomic replicons (40). For example, in both studies, data indicated that the SH-G gene junction terminated transcriptions with the greatest efficiency and produced low levels of readthrough transcripts, whereas the NS1-NS2 gene junction had the highest level of readthrough (40).

### RNA-seq analysis revealed an increase in the frequency of transition but not transversion mutations in the presence of ribavirin.

**FIG 4:**
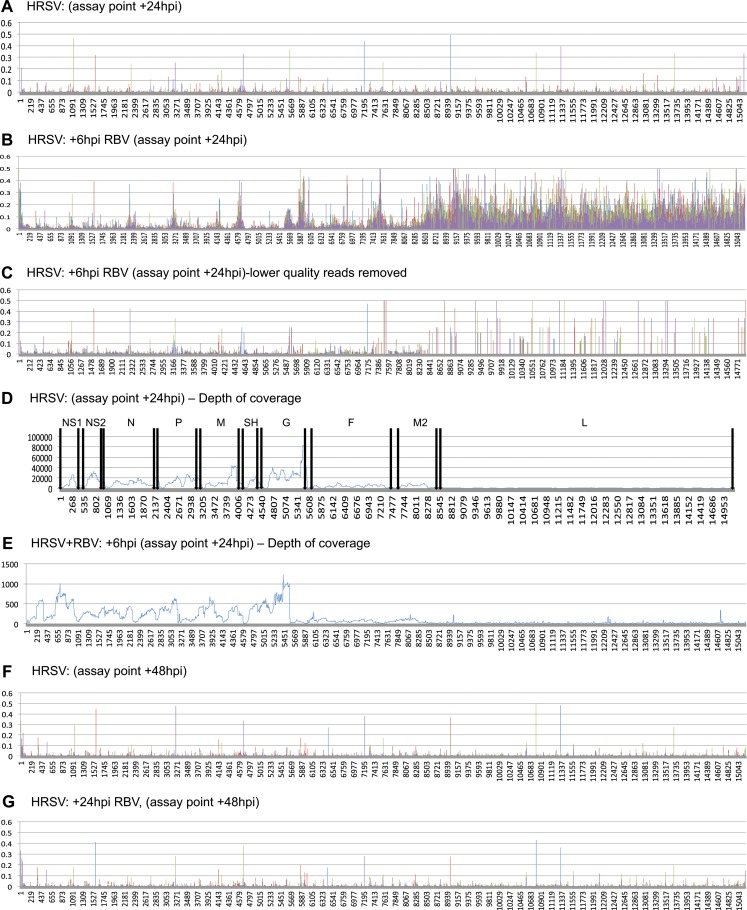
Analysis of depth of coverage and minor variation along the HRSV genome in the presence or absence of RBV. In each part, the nucleotide position is indicated along the *x* axis. Charts illustrating minor variation show the proportion of minor nucleotide calls at each nucleotide as a fraction of 1; thus, a bar height of 0.4 indicates that at that nucleotide position, some 40% of the sequence reads indicated an alternate base relative to the consensus. Charts illustrating coverage depth are simple plots of the depth of sequence coverage at each nucleotide position. (A) Locations of minor variant base calls for HRSV mRNA mapped to the HRSV genome at 24 h postinfection. (B) Locations of minor variant base calls for HRSV mRNA mapped to the HRSV genome at 24 h postinfection for infections where ribavirin was added to the medium at 6 h postinfection, using the same mapping pipeline as that used for panel A. (C) Analysis of the same raw sequence data as in panel B, but with low-quality sequence reads removed (e.g., all reads with a MAPping Quality (MAPQ) value of <11 and a mapped read length of <50 bases). (D) Depth of coverage of sequence data for HRSV mRNA mapped to the HRSV genome at 24 h postinfection, with the locations of HRSV genes indicated. (E) Depth of coverage of sequence data for HRSV mRNA mapped to the HRSV genome at 24 h postinfection for infections with ribavirin added at 6 h postinfection. Note the change in scale on the *y* axis. (F) Locations of minor variant base calls for HRSV mRNA mapped to the HRSV genome at 48 h postinfection. (G) Locations of minor variant base calls for HRSV mRNA mapped to the HRSV genome at 48 h postinfection for infections where ribavirin was added to the medium at 24 h postinfection, using the same mapping pipeline as that used for panel A.

To access the frequency of potential nucleotide substitution in the different populations, the QuasiRecomb algorithm (36) was used to obtain coverage and data on the proportion of each nucleotide used at any given location on the genome. The results were further processed by an in-house script to determine which nucleotide was the most abundant at each nucleotide position and how frequently any of the other three nucleotides were used at that position. We used a similar approach to derive consensus genomes and examine nucleotide variation that corresponded with increases in virulence of Ebola virus in a guinea pig model of infection (39), to measure Ebola virus evolution during the 2014-2015 West African outbreak (37), and to investigate infections of human and bat cells with Hendra virus (41). These data are displayed as frequencies of use of minor nucleotides along the genome (Fig. 4). For example, in the case of HRSV assayed at 24 hpi, there were 13 nucleotide positions that had a substitution in 30% or more of the sequence reads that mapped across that location (Fig. 4A). On first inspection, the nucleotide substitution frequency in HRSV RNA isolated from cells that had been treated with ribavirin at 6 hpi (assay point at 24 hpi) seemed large, especially in the L gene sequence (Fig. 4B). However, further inspection of the data revealed that given the 166-fold decrease in mappable reads at this assay point, there were relatively few quality reads, and the apparent nucleotide frequency variation decreased when lower-quality reads were removed (Fig. 4C). We also artificially reduced the number of mapped reads obtained without ribavirin to the levels seen when we examined cells treated with ribavirin at 6 hpi (data not shown). When we examined the set for minor variants, we saw a similar pattern of nucleotide variation (data not shown), confirming our supposition that compared to the larger number of mappable reads (Fig. 4D), the small number of mappable reads (Fig. 4E) led to an apparent increase in variation which cannot be attributed to ribavirin treatment.

Analysis of the sequence data obtained when ribavirin was added at 24 hpi and viral RNA was analyzed at 48 hpi provided higher-quality reads. For the control untreated HRSV infection, there were 8 nucleotide positions that had a substitution in 30% or more of the sequence reads that mapped across that location (Fig. 4F). For the HRSV infection in which ribavirin was added at 24 hpi and the nucleotide sequence was analyzed at 48 hpi, there were 5 nucleotide substitutions that were present in 30% or more of the sequence reads that mapped across that location (Fig. 4G).

**FIG 5:**
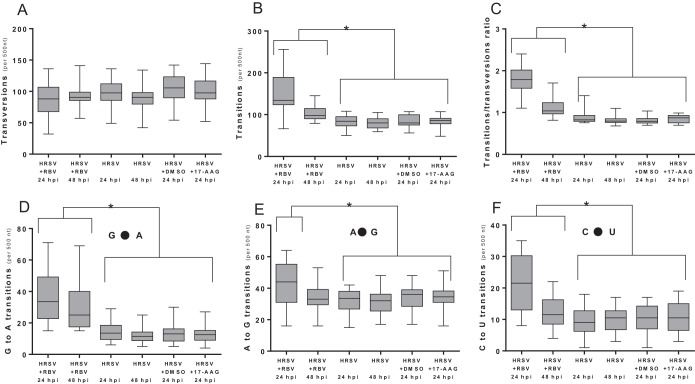
Transition and transversion analyses of each of the culture conditions. The analysis was implemented using a 500-nucleotide sliding window across the genome. The area coding for the polymerase gene was not included in the analysis because the sequencing depth for this genomic region was much lower. Each box plot indicates the mean value, the 25th and 75th percentiles, and the 95% confidence interval (CI) of the mean. For comparisons, the paired *t* test was implemented using the Prism software package (GraphPad). (A) Purine-pyrimidine transversions; (B) purine-purine and pyrimidine-pyrimidine transitions; (C) transitions and transversions; (D) G-to-A permutations (E); A-to-G permutations; (F) C-to-U permutations. The analysis was also performed using a 100-nucleotide sliding window (data not shown), and the results were similar.

As described in the introduction, ribavirin treatment might be expected to result in an increase in transition mutations specifically. To distinguish this effect, analysis of mutations was performed using Bioedit Textpad and Excel software. Initially, all transitions and transversions were scored and given a binary identification. Transversions were also analyzed as an internal control to investigate whether the frequency of any mutation increased in the presence of ribavirin. Subsequently, a sliding window of 500 nucleotides was arbitrarily chosen and was moved across the full length of the reference sequence by increments of 500 nucleotides. In each step, the numbers of transversions and transitions among the minor sequence quasispecies were determined (Fig. 5). The data indicated that transition mutations significantly increased in the presence of ribavirin compared to those in the untreated control. The frequency of transversions was not significantly different. Reflecting this, the ratio of transitions to transversions was significantly higher in ribavirin-treated infected cells than in untreated infected cells. There was more experimental noise associated with the analysis of transitions for cells treated with ribavirin at 6 hpi (assay point at 24 hpi) than for cells with ribavirin added at 24 hpi (assay point at 48 hpi). We attribute this to the lower-quality sequence reads, and the comparative data shown in Fig. 5 are based on sequence reads obtained with the more abundant transcripts produced from the first half of the genome.

**FIG 6:**
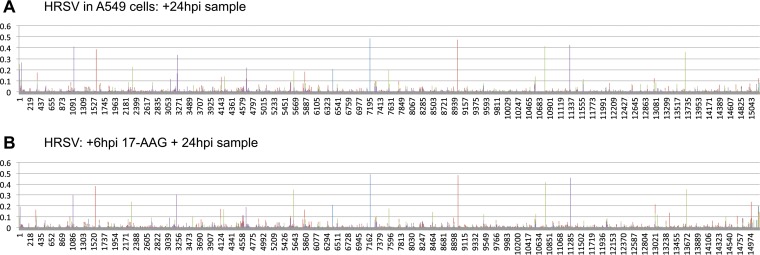
Analysis of minor variation along the HRSV genome in the presence or absence of 17-AAG. In each part, the nucleotide position is indicated along the *x* axis. As described in the legend to Fig. 4, the minor variation shows the proportion of minor nucleotide calls at each nucleotide as a fraction of 1; thus, a bar height of 0.4 indicates that at that nucleotide position, some 40% of the sequence reads indicated an alternate base relative to the consensus. (A) Locations of minor variant base calls for HRSV mRNA mapped to the HRSV genome at 24 h postinfection in A549 cells. (B) Locations of minor variant base calls for HRSV mRNA mapped to the HRSV genome at 24 h postinfection for infections where 17-AAG was added to the medium at 6 h postinfection in A549 cells, using the same mapping pipeline as that used for panel A. Both data sets were generated independently of those analyzed in Fig. 4.

These results were also compared to data obtained from cells treated with 17-(allylamino)-17-demethoxygeldanamycin (17-AAG) as a control. Treatment of infected cells with 17-AAG inhibits the chaperone activity of HSP90 and results in destabilization of the HRSV L protein (42). Thus, 17-AAG acts indirectly as an inhibitor of RdRp accumulation (rather than activity) and has been shown to have an antiviral effect on HRSV (42–44) and other negative-strand RNA viruses (45). In the present study, the nucleotide substitution frequency was analyzed at 24 hpi. At this time point, there were 8 nucleotide positions that had a substitution in 30% or more of the sequence reads that mapped across that location in the untreated control (Fig. 6). For the HRSV infection in which 17-AAG treatment was started at 6 hpi and the HRSV sequence was analyzed at 24 hpi, this resulted in a 9-fold decrease in mappable reads to the HRSV genome (Table 1); there were 9 nucleotide positions that had a substitution in 30% or more of the sequence reads that mapped across that location (Fig. 6). There was no evidence that 17-AAG caused an increase in the frequency of transitions or transversions in HRSV-infected cells (Fig. 5).

### Transition and transversion substitutions occur in clusters along HRSV RNA.

**FIG 7:**
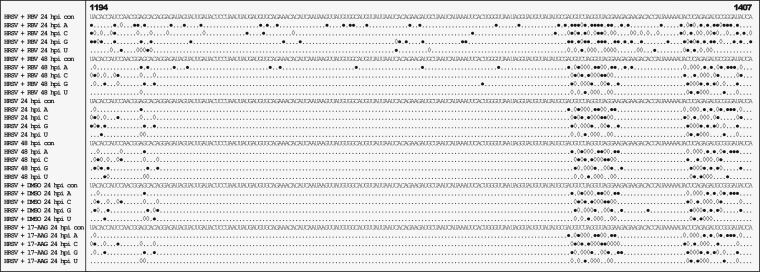
Representative analysis of transitions (full circles) and transversions (open diamonds) resulting from the minor nucleotide variants at each position of the HRSV genome with the different treatment regimens. For each condition, the consensus sequence (con) is shown on the first line. The following four lines represent each of the four nucleotides found as minor variants. The cutoff value was 0.5%, and the region of the genome shown is indicated by the bold numbers at the top.

**FIG 8:**
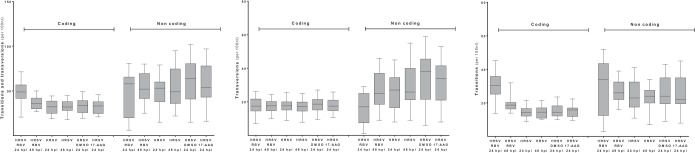
Analysis of transitions and transversions in coding and noncoding regions of the HRSV genome with the different treatment regimens.

Interestingly, analysis of the positions and frequencies of the transition and transversion mutations along the HRSV genome, for virus either left untreated or treated with ribavirin, suggested that these background substitutions did not occur with an even distribution and instead clustered in discrete hot spots (Fig. 7). These hot spots were equivalent for HRSV grown in either HEp-2 or A549 cells, indicating that this observation was not dependent on these two cell types. Some of these hot spots were located in the intergenic regions, but some were also found in coding regions. There was no significant increase in transition or transversion substitutions in HRSV-infected cells treated with DMSO (Fig. 5), which was the ribavirin solvent. However, there was an increase in the number of transition mutations between (and within) hot spots with ribavirin treatment. From this analysis, it is clear that nucleotide changes in the viral genome due to the action of ribavirin occur both in these clusters and between these clusters (Fig. 7). The data also indicate that, in general, there is a greater frequency of transition and transversion substitutions within noncoding regions than within coding regions (Fig. 8).

## DISCUSSION

For the first time, we have used RNA-seq at a high resolution to investigate HRSV RNA synthesis and to analyze the effects of a widely used and reportedly broad-spectrum antiviral drug, ribavirin, on infected-cell RNA accumulation and viral RNA mutation frequency. RNA-seq analysis revealed that in the absence of ribavirin, HRSV-specific RNAs formed up to one-third of all RNA reads, suggesting a remarkable domination of the cellular RNA pool, at least in cell culture models. Our analysis of sequencing data to generate minor variants was indeed capped at 1 million reads mapping to the HRSV genome.

Ribavirin treatment of HRSV-infected cells resulted in decreases in progeny virus and overall levels of viral RNA as determined by the number of sequence reads that mapped to the HRSV genome (Table 1). While this reduction in the number of reads mapping to the HRSV genome in proportion to the total number of reads obtained could be explained by an increase in cellular mRNA levels caused by ribavirin treatment, the data we have do not support this conclusion. This would require a significant (∼10-fold) and across-the-board increase (and in just the same genes detected in the ribavirin-only control samples) in the total amount of mRNA in the cell. This would be unusual for either ribavirin treatment or HRSV infection. In addition, we saw no evidence of increases in total RNA extracted from the cells treated solely with ribavirin.

Our analysis indicated that the frequencies of transitions, but not transversions, in the HRSV genome were significantly different between infected cells treated with ribavirin and untreated infected cells. No increase in nucleotide transitions or transversions in the HRSV sequence was associated with treatment of infected cells with 17-AAG, an inhibitor of HSP90 (Fig. 5) and HRSV polymerase accumulation and function (42). There was an approximately 1.5-fold increase in the recorded frequency of transitions on HRSV RNA in infected cells treated with ribavirin at 24 hpi compared to the untreated control cells (Fig. 5). This is less than the 4.4-fold increase in the frequency of transitions associated with the treatment of poliovirus-infected cells with 400 μM ribavirin (33), and the reasons for this are unclear. In the poliovirus study, cells were pretreated with ribavirin and the drug was maintained throughout the 6-h infection, whereas in this study ribavirin was not added to the HRSV-infected cells until either 6 or 24 hpi. Alternatively, or in addition, it might be that the HRSV RdRp is more stringent than that of poliovirus, possibly due to the 2-fold longer length of the HRSV genome. In this scenario, the HRSV RdRp might better discriminate against ribavirin triphosphate, resulting in reduced incorporation and thus a lower mutation rate. Regardless, elevations in mutation frequencies of as little as 2-fold have been proposed to lead to fitness losses and extinctions of large RNA virus populations in cell culture and animal models of infection (46), so the 1.5-fold increase in mutation frequency observed here could explain why ribavirin treatment resulted in reduced HRSV-specific RNA levels and progeny virus (Fig. 1 and 2). While these results strongly suggest that the anti-HRSV action of ribavirin is through direct incorporation into nascent RNA by the viral polymerase, we cannot exclude the possibility that other proposed activities, such as reduction of the GTP pool, also contribute to reduced viral growth and HRSV-specific RNAs, although ribavirin did not affect the overall levels of cellular mRNA (Table 1).

As noted, the read depth of viral RNA was much lower when infected cells were treated with ribavirin at 6 hpi than when infected cells were treated with ribavirin at 24 hpi. In order to compare the nucleotide substitution frequencies under these two experimental conditions, we used data from the 3′-proximal half of the HRSV genome. This is because the majority of the 5′-proximal portion of the genome encodes the L mRNA, which is the least abundant transcript and subsequently has even fewer sequence reads mapping to it. The error rate of viral RdRps is estimated to be between 1.5 × 10^−3^ bp^−1^ (bacteriophage Qβ) and 7.2 × 10^−5^ bp^−1^ (influenza virus) (47). This relaxed fidelity of RdRp activity is an important feature of RNA virus biology, providing a source of sequence diversity that can allow virus quasispecies to form, enabling the virus to adapt successfully to changing environments. However, this inherent RdRp error rate can also be detrimental to virus biology and lead to the generation of nonviable templates that reduce overall viral fitness. The term “error catastrophe” has been used to describe the outcome of an RdRp error rate at which too many nonviable templates are generated and a virus population becomes unsustainable, and it is believed that many viral RdRps operate close to this threshold (48, 49). Although the length of the HRSV genome is only ∼1.5 times longer than that of HCV or poliovirus, a 50% increase in the frequency of transition mutations appears to be sufficient to cause a loss of viable virus.

Interestingly, the analysis indicated that transitions and transversions occurred in clusters along the HRSV RNA genome (Fig. 7), and this was the same for viruses grown in different cell lines (e.g., HEp-2 and A549) and under a variety of different treatment conditions (e.g., in the presence of 17-AAG). Some of these clusters localized to the intergenic regions, and one possibility for this is the known tolerance for sequence changes within this region of the HRSV genome, as reflected in the differences between the frequencies of transition and transversion events between the coding and noncoding sequences (Fig. 8). It is worth noting that these are the positions where such minor variants can occur but that these do not reflect the average consensus sequence. Analysis indicated that treatment with ribavirin increased the frequency of transition mutations in both the coding and noncoding regions (Fig. 8).

The data also indicated a preponderance of A-to-G changes in the minor variants, which is a characteristic of adenosine deaminases acting on RNA (ADAR), a modification which, depending on the virus, has been reported to have both antiviral and proviral activities (50). Genome-wide association studies have shown an increase in ADAR transcripts during HRSV infection in a mouse model (51). Escape mutant analysis with antibodies specific for the G protein suggested that the G gene could potentially be modified by ADAR activity (52).

Analysis of the abundances of HRSV mRNAs at the two time points analyzed indicated that they did not reflect the linear gradient that is predicted by the generally accepted and long-standing model for polar and sequential transcription of negative-strand RNA viruses (Fig. 3). We used several different algorithms to analyze the RNA-seq data, and each of these indicated that the abundances of viral mRNAs in the cell at these specific time points did not follow a precise linear gradient. For the final data analysis shown in Fig. 3, we manually calculated the number of fragments per kilobase of gene per million bases mapped. Even a very conservative analysis of the RNA-seq data indicated a nonpolar abundance of viral transcripts (Fig. 3B). Given that there are very strong data to show that the HRSV RdRp cannot enter the template internally (17) and that in an *in vitro* transcription assay the amount of each transcript reflects the position of the gene on the genome (19), this result indicates that while gene order is a principle determinant of transcript abundance, it is not the only factor, with mRNA stability likely making an important contribution to relative mRNA levels. Importantly with respect to this study, ribavirin had no effect on the relative abundances of the 10 sequentially transcribed HRSV transcripts (Fig. 3). Our current findings also suggest that ribavirin has no detectable influence on RNA synthesis processes that may affect transcript stability. These include RNA processing events, such as 5′ capping and poly(A) tail addition. The RNA-seq approach may provide a bias toward the recording of different mRNA abundances, although the data indicated that the percentages of readthrough of HRSV transcripts followed the same pattern as that described using alternative approaches (40).

In a study using RNA-seq to measure viral mRNA abundance, a similar result was recently described for the analysis of Hendra virus mRNA in infected human or bat cells (41). Similar to HRSV, Hendra virus is a member of the Paramyxoviridae family, and both viruses share similar genome architectures and replication and expression strategies. The order of genes along the Hendra virus genome (3′ to 5′) is N, P, M, F, G, and L. In the analysis of either human or bat cells infected with Hendra virus, there was a steep decline in the abundance of transcripts at the M-F gene boundary, and the mRNA encoding the G protein was more abundant than the preceding mRNA, encoding the F protein (41).

In summary, RNA-seq analysis was used to investigate HRSV RNA synthesis in infected cells and cells treated with ribavirin. The data indicated that both transition and transversion mutations occurred in clusters along the HRSV genome. The frequency of transitions was increased in HRSV-infected cells treated with ribavirin and correlated with reductions in the abundance of viral RNA and in progeny virus, consistent with a loss of viral fitness.
